# Relationships of the Location and Content of Rounds to Specialty, Institution, Patient-Census, and Team Size

**DOI:** 10.1371/journal.pone.0011246

**Published:** 2010-06-21

**Authors:** James R. Priest, Sylvia Bereknyei, Kambria Hooper, Clarence H. Braddock

**Affiliations:** 1 Seattle Children's Hospital, Seattle, Washington, United States of America; 2 Department of Pediatrics, University of Washington School of Medicine, Seattle, Washington, United States of America; 3 Department of General Internal Medicine, Stanford University School of Medicine, Stanford, California, United States of America; Mount Sinai School of Medicine, United States of America

## Abstract

**Objective:**

Existing observational data describing rounds in teaching hospitals are 15 years old, predate duty-hour regulations, are limited to one institution, and do not include pediatrics. We sought to evaluate the effect of medical specialty, institution, patient-census, and team participants upon time at the bedside and education occurring on rounds.

**Methods and Participants:**

Between December of 2007 and October of 2008 we performed 51 observations at Lucile Packard Children's Hospital, Seattle Children's Hospital, Stanford University Hospital, and the University of Washington Medical Center of 35 attending physicians. We recorded minutes spent on rounds in three location and seven activity categories, members of the care team, and patient-census.

**Results:**

Results presented are means. Pediatric rounds had more participants (8.2 vs. 4.1 physicians, *p<.001*; 11.9 vs. 2.4 non-physicians, *p<.001*) who spent more minutes in hallways (96.9 min vs. 35.2 min, *p<.001*), fewer minutes at the bedside (14.6 vs. 38.2 min, *p = .01*) than internal medicine rounds. Multivariate regression modeling revealed that minutes at the bedside per patient was negatively associated with pediatrics (−2.77 adjusted bedside minutes; 95% CI −4.61 to −0.93; *p<.001*) but positively associated with the number of non-physician participants (0.12 adjusted bedside minutes per non physician participant; 95% CI 0.07 to 0.17; *p = <.001*). Education minutes on rounds was positively associated with the presence of an attending physician (2.70 adjusted education minutes; 95% CI 1.27 to 4.12; *p<.001*) and with one institution (1.39 adjusted education minutes; 95% CI 0.26 to 2.53; *p = .02*).

**Conclusions:**

Pediatricians spent less time at the bedside on rounds than internal medicine physicians due to reasons other than patient-census or the number of participants in rounds. Compared to historical data, internal medicine rounds were spent more at the bedside engaged in patient care and communication, and less upon educational activities.

## Introduction

Conducting rounds is the core activity for both patient care and learning the practice of medicine on inpatient teaching services in the United States. A minimal definition of rounds includes patient presentations by junior team members to senior team members followed by group decision-making on patient care for the day, often incorporating interactions with patients and family members [1], [2], [3].

Rounds in the teaching hospital is also a key activity in the education of medical students and housestaff. Two studies of internal medicine inpatient services in the early 1990s noted 22–29% of rounds were spent on educational activities, 47–55% on patient presentation and discussion, and only 8–12% on direct patient interactions [4], [5]. No similar comprehensive observational data exist for pediatrics outside the ICU.

The transformation of medical informatics, prevalence of chronic disease, and ACGME duty-hour restrictions have changed the practice of academic medicine since these historical observations of internal medicine were performed. In response to these changes 95% of internal medicine residency programs voluntarily employed patient-census limits as a mechanism for balancing education with institutional service and patient care^15^. The ACGME imposed admission caps in July, 2009 for internal medicine but not pediatrics [6], [7] [8]. However, the effect of patient-census, or limitations thereof, upon the education and patient care activities on rounds remains undefined.

The ACGME restriction of duty-hours in July, 2003 pressured residency programs and attending physicians to compress the competing goals of patient care and education during rounds within an inelastic period of time [9]. Recent assessments suggest largely unchanged patient outcomes under the duty-hour rules [10], [11]. However, the effects of duty-hour restriction upon trainee education on rounds are also unknown [12], [13], [14], [15].

Education and patient-care activities on rounds are closely related to the location where physicians conduct rounds. Trainees and attending physicians have consistently expressed a preference for education on rounds to occur at the bedside [16], [17], [18], [19], [20]. A small experimental literature also describes a weak preference by patients and family members for bedside rounds, citing increased time with physicians and better understanding of the care provided [21], [22]. Accordingly, many hospitals have moved towards a family-centered style of care emphasizing bedside rounds, use of lay terminology, and an interdisciplinary care team with the goal of promoting active participation of patients and their family members in the medical decision making process [23], [24], [25]. Some institutions report discharges earlier in the day following implementation of family-centered rounds, but fail to delineate changes in the patient care and teaching activities occurring on rounds [22], [23], [26].

Given changes in academic medicine, the deficiency of contemporary data from multiple institutions, and the absence of data from pediatrics we performed an observational study of rounds encompassing internal medicine and pediatrics at two academic institutions. We had two objectives for this study to better understand the current state of rounds at two academic medical centers: (1) collect observational data on the location and activities that constitute rounds while recording the participants on rounds and patient-census; and (2) perform a descriptive cross-sectional analysis of differences by specialty, institution, patient-census, and team-composition on patient care and education during rounds.

## Methods

### Data Collection

The Institutional Review Boards at both Stanford University and the University of Washington approved the study for an educational program evaluation, and each participant on the team received a written consent. We observed rounds on internal medicine and pediatric inpatient services at Stanford University School of Medicine (Stanford Hospital and Lucile Packard Children's Hospital) and University of Washington School of Medicine (University of Washington Medical Center and Seattle Children's Hospital). Each inpatient service was observed on rounds for 7 to 15 weekdays. Observations were divided between a convenience sample of housestaff teams at a given institution covering general medical patients (Table 1).

**Table 1:** Characteristics of Observations at the Four Participating Hospitals.

	*Seattle Children's Hospital*	*Lucille Packard Children's Hospital*	*University of Washington Medical Center*	*Stanford University Medical Center*
**Hospital Beds**	250	275	354	456
**General Medicine Teams**	5	2	5	6
**Housestaff Teams**	4	1	4	5
**Observed Housestaff Teams**	3	1	3	3
**Team Composition (seniors/interns/students)**	2/4/1-3	1/2/1-2	1/1/1-2	1/2/1-2
**Attending Physicians**	3-5	1-2	1	1
**Included Observations**	15	14	15	7

We defined the start of rounds as a simple majority of senior team members (attending physicians or senior residents) and junior team members (interns and students) assembled to start the group patient care, educational, or administrative activities for the day. Our definition for the end of rounds was the completion of patient care, educational, and administrative activities as the majority of team members shifted focus toward completing individual responsibilities.

We logged all participants in rounds including the number of physician participants (attending physicians, fellows, and housestaff), medical students, and non-physician participants (nurses, pharmacists, nutritionists, social workers, case managers, and other medical staff) if the individual substantively participated for any portion of rounds longer than 30 seconds, whether through verbal contribution or the simple appearance of active listening to ongoing discussions. Attending physicians were classified as generalists or subspecialists. The number of patients on the team census was documented in addition to the breakdown of new patients admitted within the previous 24 hours, and old patients on the team census for greater than 24 hours.

Based on prior studies we developed discreet mutually exclusive categories for both location of rounds and the activities occurring during rounds [4], [5], [27]. Location categories (bedside, hallways, and conference rooms) and activity categories (new patient discussion, old patient discussion, patient/family interaction, educational activities, data review, staff interaction, and other activities) are described in Table 2. Notably, education activities consisted of presentations or teaching by any team member on topics directly related or unrelated to patient care independent of location. Bedside minutes refer to location, and included any activities conducted in a patient room. The location and content of rounds was recorded in real time rounded up to the minute with a stopwatch by one of three observers (JP, SB, KH). For each minute on rounds only one activity category and one location category was recorded. We collected audio recordings of observations at Stanford Hospital, to allow all rounding content to be analyzed by only one observer (JP).

**Table 2:** Definitions of Categories Used to Record the Location and Content of Rounds.

*Locations*	*Definition*
**Conference Room**	All rounding activities conducted in work rooms, team rooms, call rooms, conference rooms, or radiology reading rooms.
**Bedside**	Includes any rounding activities conducted in a patient room
**Hallway**	Encompasses nursing stations, hallways outside of patient rooms and between wards, and stairwells.

*Content*	*Definition*
**New Patient Discussion**	Presentation or discussion of patients new to the service, i.e. patients admitted within the previous 24 hours, in-hospital transfers, and “bounce-backs”
**Old Patient Discussion**	Interval presentation or discussion of patients on the team census for greater than 24 hours.
**Patient/Family Interaction**	Direct team interaction of any kind with patients or family members including communication, counseling, teaching, examination, or direct patient care.
**Data Review**	Time spent as a team systematically reviewing patient data such as radiology rounds, lab-values, or detailed chart review.
**Staff Communication**	Communication with consultant physicians or other medical staff such as nursing, respiratory therapy, nutrition, social work, or case management.
**Education Activities**	Presentations or teaching by any team member on topics directly related or unrelated to patient care.
**Other Activities**	Encompasses all activities not explicitly mentioned above such as non-medical discussions, waiting for team members, looking for a place to round, planning or administrative tasks, and transit time between patients.

### Statistics

We used the R language and environment for statistical computing, version 2.8.1 (R Foundation for Statistical Computing, Vienna, Austria) for all statistical analysis [28]. Bedside minutes and interaction minutes were divided by patient-census, while new patient and old patient discussion were divided by the number of new and old patients respectively. We examined the mean for differences by specialty with a two-group student's *t*-test. All reported p-values are two-sided. We applied Holm's method as a simple and moderately conservative test for multiple hypothesis correction that is unaffected by dependencies between tests [29].

To gauge the predictors of patient care and educational aspects of rounds, we performed multivariable regression analysis using a generalized estimating equation (GEE) methodology for two outcome variables: bedside minutes per patient and education minutes. Both outcome variables required square root transformation to maintain a constant variance for the purposes of regression. Hereafter the transformed outcome variables are referred to as “adjusted bedside minutes” and “adjusted education minutes.” For each predictor variable we also reported a bivariate regression model. Both multivariate models incorporated institution, specialty, number each of physician, non-physician, and medical student participants, and the number of new and old patients as predictor variables. We included minutes in each of the three locations (hallway, bedside, and conference room) as predictor variables in the adjusted education minutes model. We noted the occasional absence of an attending physician during some observations of internal medicine, so we incorporated the presence of an attending physician as a binary predictor variable in each model.

Some observations were performed on consecutive workdays with the same attending physician and housestaff teams. To account for correlated observations within the multivariable model, we defined each set of attending physicians and housestaff as a group for the purposes of estimating a correlation matrix for the GEE. We used an exchangeable correlation matrix, which estimates a non-zero uniform correlation for all variable pairs within a defined group. Reported confidence intervals are based on Huber-White estimation of the standard error, and p-values derived from the Wald statistic.

## Results

### Observational Data and Comparison by Specialty

We made 56 timed observations over a 10-month period in 2007 and 2008 of 10 housestaff teams. Due to incomplete or poor quality audio recordings, 5 observations of internal medicine rounds at Stanford Hospital were unable to be scored and therefore excluded. We analyzed 51 observations that included 35 attending physicians, 82 residents, 33 medical students, and 291 patients. For purposes of data presentation, institutions are referred to anonymously as Institution A and Institution B.

Table 3 provides means, standard deviations, and statistical tests divided by specialty. When compared to internal medicine, pediatric rounds had more physician and non-physician participants who rounded on more patients. Pediatricians spent more time in the hallways and less time at the bedside on rounds while discussing old patients for more time than internal medicine physicians. Overall both specialties spent a clinically insignificant similar number of minutes performing data review, staff communication, and other activities, and had similar number of medical student participants; therefore means are presented but no statistical test was performed. Minutes spent on educational activities, new patient discussion, or patient and family interaction were statistically indistinguishable. We did observe other differences between pediatric and adult inpatient rounds; 17% of attending physicians participating in internal medicine rounds were subspecialists, compared to 62% of attending physicians on pediatric rounds. Bedside minutes, patient interaction minutes, and educational activity minutes are presented as a percentage of total minutes on rounds in Table 4.

**Table 3:** Mean Values for Team Composition, Patient load, and Duration of Location and Activities Observed on Rounds Vary by Specialty.

	Internal Medicine (n = 22 observations)		Pediatrics (n = 29 observations)		unadjusted *p*-values^a^	adjusted *p*-values^b^
**Minutes on rounds, mean (SD)**	104.8	(40.8)	124.1	(36.1)	0.09	0.44
**Patient census, mean (SD)**	8.9	(2.51)	13.9	(8.4)	**0.005**	**0.04**
**Care Team**						
Total number of physicians, mean (SD)	4.1	(2.2)	8.2	(3.7)	**<.001**	**<.001**
Total number of medical students, mean (SD)	1.5	(0.8)	1.5	(1.2)		
Total number nursing & other medical staff, mean (SD)	2.4	(1.8)	11.9	(7.0)	**<.001**	**<.001**
**Location (minutes)**						
Conference room, mean (SD)	31.3	(44.4)	12.7	(16.4)	0.07	0.44
Bedside, mean (SD)	38.2	(29.3)	14.6	(12.5)	**0.001**	**0.01**
*Bedside minutes per patient, mean (SD)*	4.8	(4.1)	1.9	(1.9)	**0.01**	**0.05**
Hallways, mean (SD)	35.2	(29.5)	96.9	(31.6)	**<.001**	**<.001**
**Content (minutes)**						
New patient discussion, mean (SD)	19.0	(29.6)	21.3	(20.9)	0.77	1.00
*New patient discussion per new patient, mean (SD)*	11.1	(10.9)	8.3	(5.1)	0.42	1.00
Old patient discussion, mean (SD)	28.5	(22.5)	53.1	(23.6)	**<.001**	**<.001**
*Old patient discussion per old patient, mean (SD)*	3.8	(2.1)	8.2	(5.3)	**<.001**	**<.001**
Patient & family interaction, mean (SD)	24.8	(16.3)	16.7	(10.3)	0.05	0.35
*Patient & family interaction per patient, mean (SD*)	3.1	(2.3)	2.0	(1.8)	0.09	0.44
Educational activities, mean (SD)	10.3	(13.3)	7.6	(8.2)	0.40	1.00
Staff communication, mean (SD)	1.8	(2.4)	2.8	(2.5)		
Data review, mean (SD)	3.2	(5.3)	2.1	(3.9)		
Other activities, mean (SD)	16.4	(11.1)	20.5	(11.0)		

a Unadjusted p-values calculated from a two group t-test.

b adjusted p-values represent the Holm correction for multiple comparisons. All p-values are two-sided.

**Bold text** indicates significance of .05 or less.

Grey box indicates no significance test was performed.

**Table 4:** Comparison of Historical Data on Rounds to Medicine and Pediatrics Observations.

	Internal Medicine (*Elliot et al.*)	Surgical & Obstetric (*Elliot et al.*)	Internal Medicine (*Miller et. al.*)	Internal Medicine	Pediatrics
**Observations**	44	25	96	22	29
**Year**	1990	1990	1992	2008	2007–08
**Mean Total Minutes**	90 min	38 min	100 min	104.8 min	124.1 min
**Percentage of rounds at the bedside**	9%	57%	11%	37%	13%
**Percentage of rounds on patient interactions**	8%	23%	12%	25%	15%
**Percentage of rounds spent on educational activities** *	29%	20%	22%	9%	6%

*Category defined by *Elliot et al.* as “discussion of diseases not directly related to patient care” and *Miller et al.* as “topic presentations”.

### Descriptive Multivariable Regression Modeling

To estimate the correlation parameters in an exchangeable correlation matrix for the GEE, we divided the 51 scored observations into 12 groups based on attending physician and housestaff team.

The multivariable model for adjusted bedside minutes detected five significant associations presented in Table 5. The specialty pediatrics (−2.77 adjusted bedside minutes; 95% CI −4.68 to −0.93; *p = .003*) and the number of new patients (−0.14 adjusted bedside minutes per new patient; 95% CI −0.23 to −0.05; *p = .003*) were both negatively associated with adjusted bedside minutes. Any additional participant in rounds was positively associated with adjusted bedside minutes, but institution did not display a statistically significant association with the outcome variable in this model.

**Table 5:** Generalized Estimating Equation for Association of Observed Characteristics of Rounds with Adjusted Bedside Minutes per Patient^a^.

	Bivariate Analysis				Multivariate Analysis			
Variables	ß	95% Confidence Intervals		*p*-Value	ß^b^	95% Confidence Intervals		*p*-Value
**Specialty: Pediatrics** c	−1.00	−1.84	−0.16	**0.02**	−2.77	−4.61	−0.93	**0.003**
**Institution: “Institution B”** d	−0.18	−1.13	0.77	0.72	−0.94	−2.79	0.90	0.32
**Care Team**								
Number of physician participants	−0.14	−0.21	−0.06	**<0.001**	0.23	0.01	0.46	**0.04**
Number of medical student participants	0.37	−0.19	0.93	0.08	0.51	0.09	0.93	**0.02**
Number of non-physician participants	−0.07	−0.10	−0.03	**<0.001**	0.12	0.07	0.17	**<.001**
Presence of an attending physician	0.02	−0.64	0.69	0.94	0.24	−0.27	0.75	0.35
**Census**								
Number of new patients	−0.05	−0.14	0.03	0.23	−0.14	−0.23	−0.05	**0.002**
Number of old patients	−0.08	−0.15	−0.01	**<0.001**	−0.08	−0.18	0.03	0.15

a Adjusted Bedside Minutes = √(bedside minutes/patient census), intercept 0.87.

b ß = Slope of the regression line adjusted for each variable and adjusted bedside minutes, expressed per unit of each variable.

c when compared to internal medicine.

d when compared to “Institution A”.

**Bold text** indicates significance of .05 or less.

Four significant associations are displayed in Table 6 for the adjusted education minutes model. The presence of an attending physician (2.70 adjusted education minutes; 95% CI 1.27 to 4.12; *p = <.001*) and “Institution B” (1.39 adjusted education minutes; 95% CI 0.26 to 2.53; *p = .02*) were each positively associated with adjusted education minutes. The number of old patients and bedside minutes each had a small but discernable negative association with adjusted education minutes.

**Table 6:** Generalized Estimating Equation for Association of Observed Characteristics of Rounds with Adjusted Education Minutes^a^.

	Bivariate Analysis				Multivariate Analysis			
Variables	ß	95% Confidence Intervals		*p*-Value	ß^b^	95% Confidence Intervals		*p*-Value
**Specialty: Pediatrics** c	0.04	−1.17	1.26	0.94	−1.43	−2.98	0.12	0.07
**Institution: “Institution B”** d	0.04	−1.35	1.44	0.52	1.39	0.26	2.53	**0.02**
**Care Team**								
Number of physician participants	0.12	−0.01	0.25	0.06	0.06	−0.12	0.24	0.51
Number of medical student participants	0.59	0.19	0.99	**<.001**	0.35	−0.08	0.79	0.11
Number of non-physician participants	0.01	−0.03	0.05	0.14	−0.04	−0.11	0.03	0.22
Presence of an attending physician	3.30	2.24	4.36	**<.001**	2.70	1.27	4.12	**<.001**
**Census**								
Number of new patients	0.18	0.00	0.36	**0.05**	−0.19	−0.48	0.09	0.18
Number of old patients	−0.07	−0.18	0.04	0.21	−0.13	−0.20	−0.06	**<.001**
**Location**								
Hallway Minutes	0.01	−0.01	0.02	0.44	0.01	0.00	0.03	0.13
Bedside Minutes	−0.02	−0.04	0.01	0.30	−0.03	−0.05	−0.01	**0.01**
Conference Room Minutes	0.02	0.01	0.04	**0.001**	0.02	0.00	0.04	0.10

a Adjusted Education Minutes = √education minutes, intercept 0.27.

b ß = Slope of the regression line adjusted for each variable and adjusted education minutes, expressed per unit of each variable.

c when compared to internal medicine.

d when compared to “Institution A”.

Bold text indicates significance of .05 or less.

## Discussion

Our relatively small, observational dataset on the location, activities, and participants in rounds showed measurable differences between pediatrics and internal medicine. When compared to internal medicine, pediatric rounds had more participants who spent less time at the bedside and more time in hallways, with more time discussing old patients. The average amount of time spent at the bedside on a per patient basis (4.1 minutes for internal medicine, 1.9 minutes for pediatrics) was significantly smaller for pediatrics.

This conspicuous difference in time at the bedside may illustrate different strategies to accomplish patient care on rounds. The most obvious purpose of more personnel is to care for more patients, and we did observe significantly more physician and non-physician participants on pediatric rounds caring for a greater patient-census. In addition to seeing more patients in a finite amount of time, a larger pediatric care team simply might not fit in the average patient room. These differences could offer clues as to why the pediatricians we observed spent more time in hallways and less time at the bedside.

A preference for education occurring at the bedside is described in decades of literature [16], [17], [18], [19], [20], and is accompanied by commentary on the substantial decrease in bedside education with each generation of trainees [30], [31], [32]. Until our observations there has been no contemporary data for comparison, which offer a snapshot of how the conduct of rounds may have changed over time (Table 4). For both specialties the fraction of rounds devoted to education in our sample was less than half of the 20% seen in older studies of medical and non-medical specialties. Our observations of internal medicine rounds showed a greater percentage of time spent at the bedside and on direct patient interactions than historical data. These observations are not directly comparable having taken place at multiple institutions with different clinical service needs. When taken as a whole, our observations of internal medicine rounds appear similar in length to historical observations at other institutions, but different in both location and content.

Though complex, the multivariable models may provide descriptive insight into our observations and reveal the relative contribution of census, personnel, and location on two important activities occurring on rounds; time at the bedside and time spent on education. The model for adjusted bedside minutes captured a positive association with each additional participant in rounds. Despite including more participants and more patients on rounds, pediatrics retained a strong independent negative association to adjusted bedside minutes in the multivariable model. This could indicate that pediatricians spend less time on rounds at the bedside due to reasons other than increased census and number of participants noted above. Thus our model suggests that the difference between specialties in the amount of time spent at the bedside may not be fully captured in our dataset.

Interestingly, in our model for adjusted education minutes the bedside minutes predictor displayed a negative association. This finding could suggest that in our observational sample, education on rounds occurred away from the bedside, and consequently may not be focused on physical diagnosis or inclusive of patient participation. It could also reflect observer bias in preferential recognition of education in didactic activities done in the conference room over activities occurring at the bedside, or a bias in our scoring methodology for educational activities lasting greater than 30 seconds.

There was a positive association with adjusted education minutes in our multivariable model by institution. This general observation could conceivably reflect institutional culture in such diverse areas as the selection and promotion of clinical faculty, to the structure of the inpatient teams, to the unique competencies of the attending physicians and housestaff teams that we observed. Alternatively, the difference may be spurious, reflecting the relatively small number of attending physicians observed at both institutions. The positive association between the presence of an attending physician and increase in education could suggest that attending physicians both directly teach as well as offer opportunities to facilitate educational activities. Nonetheless our data showed a measureable institutional and attending influence on the education occurring on rounds.

Our observations were of dedicated combined work and teaching rounds specific to the two included institutions, but may not be generally applicable to the structure and conduct of rounds at other institutions. The application of clinical practice patterns based on established evidence varies significantly between institutions [33]. Thus, it seems intuitive that teaching services, which have each been constructed to meet a specific clinical service requirement, are as different and unique as the clinical service needs of their host institutions. In the context of the 195 residency programs in pediatrics and 381 residency programs in internal medicine within the United States our observations at two medical schools are best viewed as hypothesis generating [34]. Future studies would sample more institutions from a variety of geographic regions to yield more generalizable results.

Finally, in our effort to better understand our dataset we employed well accepted but complex techniques of multivariable modeling which themselves can introduce opportunities for error or misinterpretation. We cannot completely exclude this possibility. During the course of the study, we observed teams over a period of days resulting in overlapping personnel and patient-census. Though we accounted for repeated measures in our statistical methods, it remains possible that the differences between specialties and significant predictors in our models reflect an artificially inflated population size skewed towards repeated observations.

The cautious interpretation of our observations and analysis within the larger context of rounds in the academic institution illustrates the tension between patient care and education. Residency programs walk a fine line in balancing the educational requirements of their housestaff with the service needs of their host institutions. The assumption by academic physicians that time at the bedside interacting with patients has a positive effect upon patient care and trainee education is the foundation of how the trainee experience on rounds is structured.

For internal medicine, the amount of time at the bedside on rounds in our sample appears higher than historical data from other institutions gathered prior to the employment of patient census caps or work hour limitations. Though our observations were carried out prior to patient-census limitations from the ACGME, voluntary census caps were in place at both observed internal medicine programs. There were no such formal limitations on patient-census in the pediatrics programs observed [6], [7] [8]. In our small observational study, aspects of increased patient-census did exert small but statistically significant negative effects on both time on rounds at the bedside and on education. The inverse relationship between patient-census and both bedside and education minutes may validate the utility of limiting patient-census as a mechanism for protecting patient care and teaching activities on rounds particularly in pediatrics where the practice is currently uncommon.

Though the comparison is not direct, we observed less education on rounds than historical observations (Table 6). Intuitively, the presence of an attending physician is most appropriate for the purposes of patient care in addition to education. Despite the slight negative association between bedside minutes and adjusted education minutes, few would advocate an increase in time away from the bedside for the purposes of education. Therefore efforts to increase education on rounds might better focus on the integration of teaching into time at the bedside. Evidence suggests that development programs focused on improving clinical teaching are popular amongst faculty, but are often short in duration and fail to sufficiently define and measure outcomes [35]. In light of the institutional influence on education captured in our multivariable model, concrete efforts at improving institutional support for teaching and directly engaging hospitalists in the academic mission are perhaps more promising solutions [36], [37], [38].

In our small observational study, pediatricians spent less time at the bedside on rounds than internal medicine physicians even when adjusting for a greater patient census and more participants in a multivariate model. Minutes spent on education was significantly associated with both the institution and the presence of an attending physician. Both bedside and education minutes were negatively associated with aspects of patient-census, which may support limiting patient-census for the purposes of protecting patient care and education. Compared to historical data, internal medicine rounds spent more time at the bedside engaged in patient care and communication, and less time on education activities. These results support further inquiry into the factors impacting patient care and education occurring on inpatient rounds.
